# Our Contemporaries

**Published:** 1913-07

**Authors:** 


### OUR CONTEMPORARIES

The *International Hospital Record* for April quotes our editorial on the Guarantee of Interneships, published in the same month, in full.

---

*Collier's* recently published a horrible story of white slavery of a bigamous wife who earned $80 a week in a 50-cent house. The same story, published as fiction, would be considered by many readers to involve an incredible degree of villainy on the part of the husband, a purely imaginative system of securing white slaves, on account of the elaborate and expensive methods employed, and a degree of subservience and physical endurance on the part of the woman impossible in these days.

---

The neglected side of the problem.

Who?

To the Editor (of the *Survey*):

(On seeing the White Slave by Abastenia St. Leger Eberle.)

This is the man that sells women's souls into hell fire. Who is the buyer? LOUISE W. KNEELAND.

This is a terrible arraignment. Ponder over it.

